# The design of a randomised controlled trial to evaluate the (cost-) effectiveness of the posterolateral versus the direct anterior approach for THA (POLADA – trial)

**DOI:** 10.1186/s12891-016-1322-2

**Published:** 2016-11-15

**Authors:** K. Rykov, I. H. F. Reininga, B. A. S. Knobben, M. S. Sietsma, B. L. E. F. ten Have

**Affiliations:** 1 Department of Orthopaedic surgery, Martini Hospital Groningen, Groningen, The Netherlands; 2 Department of Trauma surgery, University of Groningen, University Medical Center Groningen, Groningen, The Netherlands

## Abstract

**Background:**

Total hip arthroplasty (THA) is one of the most successful orthopaedic procedures. Because of the increasing number of THAs, a growing demand for faster recovery and a greater emphasis on cost-effectiveness, minimally invasive THAs have been introduced in the last decades. The direct anterior approach is a minimally invasive, tissue-sparing approach in which intermuscular planes are used. Theoretically, this approach should result in a faster recovery of physical functioning and higher health-related quality of life.

**Methods/design:**

A randomised controlled trial will be performed. Patients will be randomly allocated to undergo THA by means of the anterior or posterolateral approach. Both the intervention and control group will consist of two subgroups: 1) patients with a good bone stock who will receive an uncemented femoral stem, and 2) patients with a poor bone stock who will receive a cemented femoral stem. Patients between 18 and 90 years with primary or secondary osteoarthritis will be included. Physical functioning and health-related quality of life will be assessed by means of questionnaires. Additionally, performance based tests will be performed to objectively assess the physical functioning. Cost-effectiveness will be assessed by obtaining data on medical costs in and outside the hospital and other nonmedical costs. Measurements will take place preoperatively, two and six weeks, three months and one year postoperatively.

**Discussion:**

There is some evidence that the anterior approach results in reduced tissue damage and faster recovery in the direct postoperative period, compared to the posterolateral approach. However, there is still a lack of well-designed studies that have confirmed the better outcomes and cost-effectiveness of the anterior approach. Therefore, the purpose of this study is to assess the physical functioning, health related quality of life and the cost-effectiveness of the anterior approach, compared to the conventional posterolateral approach.

**Trial registration:**

Netherlands Trial Registry, number 5343 (registration date April 12, 2015)

**Electronic supplementary material:**

The online version of this article (doi:10.1186/s12891-016-1322-2) contains supplementary material, which is available to authorized users.

## Background

Total hip arthroplasty (THA) is considered to be one of the most successful orthopaedic interventions of the past 40 years, with 10-year survival rates exceeding 90% [1, 2]. The number of THAs has increased rapidly during the last decade, because of ageing of Western societies and an increase of the incidence of obesity. Additionally, THA is nowadays not only performed in elderly patients, but also in younger patients who are still members of the working population. Driven by this growing demand for THA, together with a greater emphasis on cost-effectiveness in health care and patients’ higher expectations of shorter hospital stays and faster recovery, alternative surgical procedures have been developed to improve the success of THA. Minimally invasive total hip arthroplasty (MIS THA) is one of these developments.

MIS THA aims at minimising damage to soft tissues during surgery in order to enhance postoperative recovery and, consequently, accelerate the return to normal daily functioning [3]. Despite the increase in use of MIS THA, its risks and benefits are still an ongoing debate in the orthopaedic community. There is a wide variety of MIS THA procedures, which have shown variable results [4]. However, these variable results for MIS THA can for a large part be contributed to the fact that some of these so-called minimally invasive techniques, are actually not minimally invasive. There are patent differences between using an alternate surgical approach intended to gain access to the hip joint through less soft-tissue dissection and using intermuscular planes (i.e. a tissue-sparing approach). Hence, when one looks critically at the literature on MIS THA, it appears that the term “minimally invasive” is often used for a conventional THA technique performed through a smaller skin incision, with at least the same amount of tissue damage under the skin compared to the conventional approach (i.e. mini-incision THA) [5].

The anterior approach for THA is a minimally invasive, tissue-sparing technique. Conceptually, the anterior approach should cause less tissue damage compared to the conventional posterolateral approach, as intermuscular planes are used without muscle dissection [6]. Moreover, by (partially) dissecting several muscles around the hip, the posterolateral approach is more prone for instability postoperatively. Whereas with the anterior approach, the musculature for pelvic stabilisation remains undisturbed [7]. These muscles also play an important role in gait function. Hence, theoretically, the anterior approach should result in a faster recovery of physical functioning and, thus, a higher health-related quality of life.

Little research has been conducted so far on the implications of MIS THA, and especially the direct anterior approach (DAA) for THA, in elderly patients [8]. The skeletal musculature of elderly patients possesses a higher vulnerability and a reduced regenerative capacity [9]. Additionally, elderly patients are characterized by a higher incidence of postoperative fatty muscle atrophy, which is associated with reduced muscular function [9]. More importantly, patients who underwent THA through a conventional approach had a significantly higher grade of muscle atrophy compared to older patients following MIS THA. Muller et al. [9] concluded that especially older patients seem to benefit from a minimally invasive approach. Whether the previously mentioned results are also applicable for the anterior approach, is still unknown.

The ability to ambulate independently is an important hospital discharge criterion since a proper gait function is crucial to perform several activities of daily living independently. Thus far, there are no studies that compare the cost-effectiveness of the direct anterior approach. Despite this, there is some evidence that accelerated postoperative care can be cost saving [10]. In this respect the anterior approach, because of its muscle sparing character and potentially faster recovery, might not only be a clinically effective but also a cost-effective surgical technique for THA.

There is however a lack of well-designed studies, and thus of objective evidence, on the effectiveness of the anterior approach. The scarce, but promising evidence that is available is derived from retrospective case series or small prospective trials [11–14]. Little is also known about whether older patients even benefit more from the anterior approach. Moreover, little information is present about the cost-effectiveness of the anterior approach for THA. Hence, the aim of this study is to conduct a randomised controlled trial to determine the clinical and cost-effectiveness of the direct anterior approach compared with the conventional posterolateral approach for THA.

## Methods/Design

### Study design

A prospective randomised controlled trial will be executed. Patients will be randomly allocated to undergo THA by means of the direct anterior approach or the posterolateral approach.

### Study population and recruitment procedure

Patients will visit the outpatient clinic at the Department of Orthopaedic Surgery at the Martini Hospital Groningen where they will receive oral and written information about the study. After screening according to the in- and exclusion criteria (Table 1). Patients with a previous surgery of the ipsilateral hip, complaints or a hip prosthesis of the contralateral hip and symptomatic osteoarthritis of the knee will be excluded, because these factors may influence the gait analysis measurements and the questionnaires when there are complaints or limitations in functioning because of these factors. Pfirrmann et al. showed that patients with symptomatic complaints after THA have more muscle atrophy [15]. Furthermore, evidence exists that atrophic muscle might not regenerate postoperatively and these factors are important in the assessment of physical functioning [16]. Moreover, because patients of 90 years and above have other comorbidities and are usually not operated in our hospital, we will exclude these patients.

**Table 1:** In- and exclusion criteria

Inclusion criteria	Exclusion criteria
- Age between 18–90 years; - Indication for THA is primary or secondary symptomatic osteoarthritis	- A history of previous surgery on the ipsilateral hip; - complaints of the contralateral hip; - a hip prosthesis at the contralateral side; - symptomatic osteoarthritis of the knee; - peripheral neuropathy; - (active) arthritis (e.g. rheumatic disease); - a history of CVA; - COPD GOLD III or IV - NYHA class III or IV - cognitive impairments. - not able to fill in questionnaires in the Dutch language

*Abbreviations: THA* Total Hip Arthroplasty, *CVA* Cerebrovascular Accident, *COPD* Chronic Obstructive Pulmonary Disease, *NYHA* New York Heart Association

After the patients have had a consideration period of two weeks, they will be included in the study. They will then be randomised into either the control group (posterolateral approach) or the intervention group (direct anterior approach) by random allocation. Both the intervention and control group will consist of two subgroups: a subgroup with patients with a good bone stock that will receive an uncemented femoral stem and a subgroup of patients, with less good bone stock that will receive a cemented femoral stem. The quality of the bone stock will be determined by routine preoperative pelvic X-rays. Since there are no studies that describe characteristics on which the type of femoral component can be chosen on pelvic X-rays, the quality of the bone stock will be determined by the orthopaedic surgeons. The main determinant will be the thickness of the femoral cortices. Because bone mineral density, and thus bone quality, decreases with age, this will also be a determinant in the choice for the stem component. Generally, patients with an age of 70 years or more will receive a cemented femoral stem. The age of 70 years is chosen because of the lower revision rates and cost-effectiveness with cemented femoral stems in this age group [17]. However, this cut-off point remains arbitrary since the life span of the older population can give misleading results on revision rates. Also, because of the longer life span of the younger population, a revision of the femoral because of aseptic loosening, will be less demanding with an uncemented femoral stem.

Two random allocation sets (for the uncemented femoral component and for the cemented femoral component) of the type of THA approach will be generated by means of a computer by an independent person (research coordinator). These allocations are then sealed in consecutively numbered opaque envelopes. Once the patient has given consent to be included in the trial, the THA approach is then randomly assigned by opening the next sealed envelope, for either the uncemented or the cemented femoral stem. After that, the envelope with the matching study number is opened by an independent research nurse who thus is blinded regarding the randomisation sequence. It is not possible to blind the patient for the allocated surgical technique, since the surgical incision site of the studied approaches is different (i.e. on the anterior or posterolateral side of the hip joint).

It is expected that around 30% of the patients are ineligible or refuse to participate in the study. Hence, approximately an inclusion period of 24 months will be needed to include 260 patients.

### Interventions

The orthopaedic surgeons who are participating in this study (*N* = 3) are experienced in performing both the posterolateral and the direct anterior approach. The learning curve for the anterior approach is said to be between the 20 and 100 cases [18–21]. The three orthopaedic surgeons have reached a number beyond the 100 cases and they will perform, or supervise, all THAs in the present study. The anaesthetic, analgesic and postoperative physical therapy protocols will be standardized. Discharge criteria will also be the same in both study groups (Additional file 1).

The type of femoral component that will be placed will be based on the quality of the bone stock. Patients with good firm bone stock of the proximal femur will receive an uncemented stem (Taperloc™, Biomet Corporation, The Netherlands), that will be hammered into the shaft of the proximal femur. Patients with less firm bone stock of the proximal femur will not receive an uncemented stem, because of the risk of periprosthetic fractures during stem insertion. They will get a cemented stem (Stanmore™, Biomet Corporation, The Netherlands). In all groups, a cemented acetabular component (Stanmore™, Biomet Corporation, The Netherlands) will be placed.

#### Intervention group – direct anterior approach

Advantage of the anterior approach is the possibility of using intermuscular planes, avoiding muscle damage by cutting or detaching muscles. The patient is placed in a supine decubitus position. The skin incision is made over, and in the direction of the lateral part of the femoral head and neck. After division of skin and subcutis, the interval between the tensor fasciae latae muscle and the sartorius muscle is identified and the overlying fascia is opened. In this part of the operation care must be taken to avoid damaging the lateral cutaneous nerve, supplying the sensation of the skin on the lateral part of the thigh. The intermuscular plane between the tensor fasciae latae and sartorius muscle is developed further down to the hip capsule. Subsequently the hip capsule is opened, allowing access to the hip joint. Preparation of the hip for implantation of a hip prosthesis can take place now, by in situ performance of the femoral neck osteotomy, removal of the femoral head and reaming of the acetabulum. Next, bone cement (Palacos®, Heraeus Medical, The Netherlands) is pressurized into the acetabular cavity, followed by insertion of the acetabular cup. After reaming of the femur, the femoral component can be placed with or without bone cement, followed by placement of a head on the femoral component, repositioning of the joint and closure in layers. In case of a cemented femoral component, bone cement is pressurized into the femoral cavity before the femoral component of the hip prosthesis is placed.

#### Control group – posterolateral approach

The patient is placed in a lateral decubitus position. The skin incision is made over the greater trochanter to cranial, with a slight curve to posterior. After transection of the subcutis, the fascia latae and the gluteus maximus muscles are split. Next, the short external rotators, namely the piriformis, the inferior and superior gemellus and the obturator internus muscles, are cut at the level of their insertion at the greater trochanter, so this approach is not muscle-sparing. In this phase of the procedure, caution is advised with the sciatic nerve, the main nerve for the lower leg. After retraction of the short external rotators backwards, the hip capsule becomes visible and can be incised, allowing access to the hip joint. Subsequently, the hip joint is then dislocated and the osteotomy of the femoral neck is performed, followed by the removing of the femoral head. The rest of the operation will essentially take place in the same manner as the anterior approach.

### Measurements

**Fig. 1:**
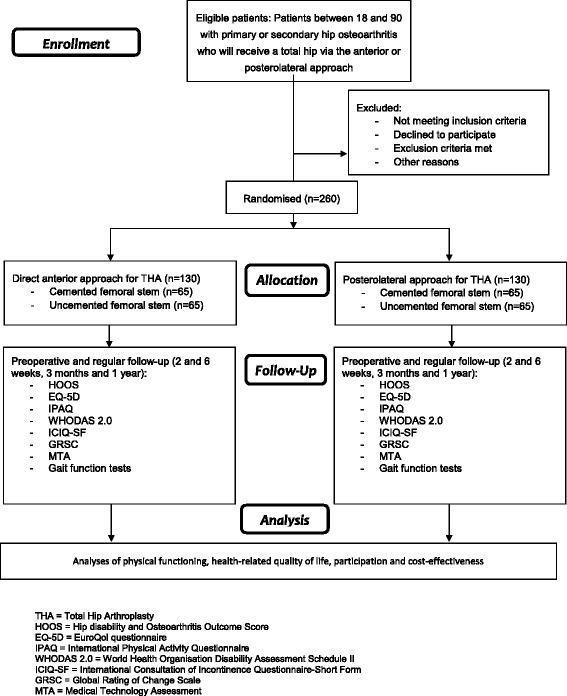
CONSORT flow chart

Measurements will be performed by an independent investigator. Standard measurement will take place preoperatively, and six weeks, three months and one year postoperatively. As it is hypothesised that the anterior approach is superior to the posterolateral approach in terms of a faster recovery, mainly in the early recovery period (shorter hospital stay, faster return to normal ADL functioning), extra measurements of physical function, gait analysis and economic evaluation will take place two weeks postoperatively. The questionnaire will be sent to the patients a few days prior to the visit or – when patient do not have an email – will be filled in during the clinical outpatient visit. The filling in of the questionnaire will approximately take 30 to 45 min of patients’ time. A CONSORT flow chart is provided in Fig. 1. Demographic data, preoperative diagnosis, height, weight and Body Mass Index (BMI), ASA classification and the Kellgren and Lawrence (KL) score will be recorded preoperatively.

#### Perioperative measurements

Surgical time, rate of complications, position of the femoral and acetabular components and length of hospital stay will be recorded. Position of the femoral and acetabular components will be measured on an anterioposterior (AP) pelvic X-ray. Inclination of the acetabular component will be measured by defining the angle between the tear drop line and the cup. The version of the cup will be measured by using the method described by Lewinnek et al. [22]. Both the cup version and inclination have been proven to have a high inter- and intraobserver reliability and have been proven to be a reliable and valid method when compared to the gold standard. [23–25] Varus and valgus alignment of the stem will be measured by taking the angle between the femoral shaft and the prosthetic component [26]. These measurements will be performed by KR and an independent orthopaedic surgeon. Measurements will be performed on standard outpatient visits six weeks and one year postoperatively. Additionally, amount of blood loss will be calculated from the drop in postoperative blood hematocrit levels, according to the formula of Nadler et al. [27].

#### Physical functioning and health-related quality of life

Main study parameter is the Patient Acceptable Symptom State (PASS), which is an approach to measure patient’s responses to treatment. The concept of PASS is based on wellbeing or satisfaction with the actual symptoms and is defined as the value on a patient-reported outcome measure (PROM) beyond which patients consider themselves well [28, 29]. The use of PASS is therefore a simple way to determine whether a patient has achieved therapeutic success. PROMs estimate the effectiveness of healthcare delivered to patients as perceived by the patients themselves. The PASS score is determined by assessing the score on a PROM of patients who are satisfied with their actual physical state and symptoms. Based on patients’ satisfaction with symptoms, the optimal cut-off value of the used PROM can be determined [30–32]. In this study, PASS scores will be derived from scores on the Hip disabilities and Osteoarthritis Outcome Score (HOOS) [33], and they will be determined for the measurements made at two and six weeks, three months and one year postoperatively. The HOOS consists of 5 subscales; pain, other symptoms, function in daily living, function in sport and recreation, and hip-related quality of life. The last week is taken into consideration when answering the questions. The Dutch version of the HOOS is considered valid and reliable [33]. Additionally, the patients’ opinion about “actual satisfaction with their symptoms regarding their hip problem” will be asked with one question. This question will be “if you spend the rest of your life with the hip symptoms you have now, how would you feel?” A Likert version of this question will be used with four response levels: very satisfied, somewhat satisfied, somewhat dissatisfied and very dissatisfied [29, 30, 32]. The scores on this question are then used to calculate PASS. The PASS scores at the previous mentioned time points will each be compared with the preoperative state and then compared between the two approaches [30].

The EuroQol questionnaire (EQ-5D) will also be administered. The health outcomes of the two approaches for THA will be assessed in terms of quality adjusted life years (QALYs). This reflects any differences in health-related quality of life based on the patients’ responses to the EQ-5D questionnaire preoperatively and at up to three points after hospital discharge (six weeks, three months and one year postoperatively). The EQ-5D is a generic questionnaire and consists of 5 questions regarding health-related quality of life [34]. The EQ-5D will also be used as a preference-based measure of health status for the cost-effectiveness analysis [35].

Next to the previous mentioned questionnaires the global rating of change will be assessed [36]. A global rating of change is a single item with which the overall (global) perceived improvement in physical functioning following THA can be assessed. This question is scored on a 7-point likert scale (1 = completely recovered; 2 = much improved; 3 = slightly improved; 4 = not changed; 5 = slightly deteriorated; 6 = much deteriorated; 7 = worse than ever), and asks for the change in physical functioning following THA.

The International Consultation of Incontinence Questionnaire-Short Form (ICIQ-SF) will be administered to evaluate whether THA has influence on urine incontinence [37]. The ICIQ-SF is a subjective questionnaire and is a recommended method to assess the grade of urine incontinence [38]. It consists of four questions on the frequency and volume of urine incontinence and its influence on daily life [37]. With the posterolateral approach the short external rotators of the hip are detached of the greater trochanter. Since the obturator internus muscle is connected to the pelvic floor musculature detachment of this muscle may have a negative effect to urine continence [39, 40], whereas with the anterior approach the pelvic floor remains undisturbed.

Physical activity behaviour will be assessed by means of the International Physical Activity Questionnaire (IPAQ) [41]. IPAQ consists of 27 items which are pre-structured into five subcategories: 1) job-related physical activity; 2) transportation-related physical activity; 3) housework, house maintenance, and caring for family; 4) recreation, sports and leisure-time physical activity; and 5) time spent sitting. The IPAQ records the activity of four intensity levels: 1) vigorous-intensity activity such as aerobics, 2) moderate-intensity activity such as leisure cycling, 3) walking, and 4) sitting. The Dutch version of the IPAQ is considered valid and reliable [42].

#### Gait function

As walking is by far the most important aspect of functional status, physical functioning will be objectively measured by means of gait analysis. The Timed Up and Go (TUG) test, the 4x10m self-paced walk test (SPWT), the stair-climb test (SCT) and the 30-s chair stand test (30s-CST) will be used to assess the physical performance. The TUG test measures the time needed for the patient to stand up from a chair, walk 3 m, turn, walk back to the chair and sit down. This test has been used to assess the functional activity [43]. For the SPWT the patient has to walk 10 m in a paced, but comfortable way and repeat this four times, for a total of distance 40 m [44]. The time the patient needs to cover this distance is measured, and the mean speed is calculated. For the SCT the patient is asked to ascend and then descend a stairs with 10 steps [45]. Finally, for the 30s-CST, the patient is asked to stand up and sit down in a chair as many time as possible. All four tests have been suggested as reliable and valid measures to compare outcomes after THA, and they are the recommended performance-based tests to assess the physical functioning before and after THA [45, 46].

#### Participation

To assess social participation, the subscale *Participation* of the World Health Organisation Disability Assessment Schedule II (WHODAS II) questionnaire [47, 48] will be assessed six weeks, three months and one year postoperatively. This subscale of the WHODAS II consists of 10 items asking about problems with participation in society. All questions are scored on a 5-item Likert scale ranging from no problem to extreme problems/cannot do. The last 30 days are taken into consideration when answering the questions. The Dutch version of the WHODAS II is considered valid and reliable [48].

#### Economic evaluation

An economic evaluation will be conducted to gain insight into cost-effectiveness of the anterior approach compared to the posterolateral approach for THA. After completion of the data collection, the health status, as determined by means of the EQ-5D, will be related to costs in additional economic analyses. These analyses will provide information on the probable cost-effectiveness of the anterior approach compared to the posterolateral approach for THA in the Dutch healthcare system.

The evaluation will be performed from a societal perspective; costs within and outside the healthcare sector will be included. Costs will be registered prospectively, i.e. within the framework of the current study, for all the included patients. A standardised questionnaire on medical costs after THA will be administered at two and six weeks, three months and one year postoperatively. The questionnaire is set up by the Medical Technology Assessment (MTA) and is used in the cost-effectiveness analyses. It contains 11 questions on indirect medical and non-medical issues regarding the THA outside of the hospital. Medical questions include hospital readmissions, visits to the hospital and/or other (paramedical) healthcare workers. Non-medical questions concern patients’ living and working situation.

The direct medical costs, such as the use of the operative theatre, hospital admission and additional interventions (e.g. blood transfusions) will be compared between the two approaches. Most salient cost advantages of the anterior approach are expected in the area of hospitalization costs. Of the various nonmedical costs, informal care costs are assumed to be substantial in the targeted population and will be registered in detail (e.g. visits to the general practitioner, physical therapy). In order to facilitate comparisons with other economic evaluations, unit prices (i.e. the price of one unit of each included cost type) are based on Dutch standard prices [49]. True costs of used resources will be estimated when standard prices are not available.

### Sample size calculation

Escobar et al. [30] showed that 70% of patients achieved an acceptable symptom state (PASS) at three months following THA, which means they have a positive PASS. The hypothesis is that the anterior approach results in a larger proportion of patients with a positive PASS at three months following THA. A difference of 20% in the proportion of patients with a positive PASS between the anterior approach and the posterolateral approach is considered clinically significant, as is described is described in the OMERACT-OARSI criteria [50]. Hence, based on the results of Escobar et al., a sample size of 60 patients in each study subgroup is needed to detect this difference of 20%, with a power of 80% and an alpha of 5%. Hence, each arm of the RCT needs to contain 120 patients. Based on previous experience, it is expected that approximately 10% will be lost in the follow-up. Hence, the total sample size will be set at 260 patients.

### Statistical analysis

To analyse the data SPSS (Statistical Package for the Social Sciences, Chicago, Illinois) will be used. A *p*-value of < .05 will be considered to indicate statistical significance. Descriptive statistics (means and standard deviations) will be used to describe the subject characteristics and the results of the groups. The PASS score is determined by assessing the score on the HOOS of patients who are satisfied with their actual physical state and symptoms. PASS scores will be determined for the measurements made at six weeks, three months and one year postoperatively. The satisfaction question, which is asked on a four-response Likert scale, will be dichotomized into “satisfied” (i.e. the patients who are “very satisfied” or “somewhat satisfied”) and “unsatisfied” (i.e. the patients who are “somewhat dissatisfied” or “very dissatisfied”) [30, 32]. This dichotomized variable will then be used as anchor to calculate cut-off values on the HOOS to determine the PASS score. Next, a ROC analysis will be conducted, using the satisified/dissatisfied variable as anchor. As optimal cut-off value will be the one that maximizes the sum of sensitivity and specificity [30].

After that, all patients will be categorized as responders (i.e. a score on the HOOS beyond the PASS score) and non-responders. Chi-square tests will be used to determine potential differences in the proportion of responders between the study and control group at the above-stated time-points. Additionally, odds ratios with corresponding 95% confidence interval (CI) will be calculated. This ratio represents the odds of being a “responder” for the study group, compared to the control group. An odds ratio larger than 1 favours the study group and the point estimate of the odds ratio is considered to be statistically significant if the 95% CI does not include the value of 1.

Changes in physical activity, participation and gait function will be analyzed with Generalized Estimating Equations (GEE) analyses [51]. Analysis of Variance (ANOVA) will be used to assess differences in urine incontinence (ICIQ-SF), radiographic measurements, surgical time, blood loss and length of hospital stay between the study and control group. Differences in complication rates will be assessed by means of a Chi-square test.

For both the study and control group the mean costs, mean effects (measured with HOOS) and mean QALY (based on the utility scores of the EQ-5D) will be calculated. Next, mean costs, mean effects and mean QALYs are used to calculate the incremental costeffectiveness ratio (ICER) [52] by dividing the difference in costs by the difference in effects/QALYs between the study and control group. Uncertainty surrounding the calculated ICER will be examined by the bootstrap method. Incremental cost-effectiveness ratios will be calculated for each of the bootstrap iterations (5000 in the present study), which then will be combined to form a mean ICER with corresponding 95% CI.

## Discussion

THA can be performed through several different approaches to the hip joint. These approaches include the anterior, anterolateral, direct lateral, transtrochanteric, posterolateral, and posterior approach. The posterolateral approach is the most commonly used approach [53].

In the last decade minimal invasive surgery techniques for THA have been introduced with the goal of reducing tissue trauma, shorten the length of hospital stay and enhance faster recovery. In the literature minimally invasive techniques are described by an incision that is less than 10 cm in length. However, some of these techniques still cause tissue damage because of soft-tissue dissection [5]. The direct anterior approach on the other hand is a tissue-sparing approach in which intermuscular planes are used to get to the hip joint. Theoretically the anterior approach should result in less tissue damage when compared to the posterolateral approach in which several of the muscles are being dissected. Additionally, because the pelvic musculature remains undisturbed with the anterior approach, it should result in a faster postoperative recovery.

Opponents of the anterior approach claim that it is a more technically demanding approach, which results in a longer operative time, more blood loss and an increase in complications during surgery [54–56]. Moreover, it is said that the anterior approach has a long learning curve, ranging from 20 up to 100 patients [18–21]. However, with more experience a decrease in blood loss, operative time and number of complications is to be expected [19–21]. In the current study all three orthopaedic surgeons are far beyond their learning curve.

The importance of the pelvic musculature in THA patients has been shown in a study by Pfirrmann et al. [15]. In their MRI study they found that patients with persistent symptoms of the operated joint had more defects of the gluteal musculature. Bremer et al. found that the direct anterior approach resulted in less soft-tissue damage to the abductors on MR images, compared to the transgluteal approach [57]. Moreover, Mayr et al. found that the gait function improved in the direct anterior group in the direct postoperative period [12]. Lugade et al. found comparable results [58]. These findings suggest that the direct anterior approach results in a faster recovery. However, these promising results are contradicted in other studies [59, 60].

There is still an ongoing debate within the orthopaedic community about the direct anterior approach for THA. This is partly due to the lack of well-designed prospective, randomized trials comparing the direct anterior with the most commonly used posterolateral approach. Moreover, there is a lack of literature about the cost-effectiveness of this tissue-sparing approach for THA. Therefore, the aim of this article is to compare the (cost-) effectiveness of the direct anterior approach with the posterolateral approach, with the hypothesis that the direct anterior approach will lead to faster recovery and a decrease in costs.

## Additional file

Additional file 1: Fast-track protocol. (DOCX 14 kb)

## Abbreviations

30s-CST: 30-second chair sand test
ADL: Activities of daily living
ANOVA: Analysis of variance
AP: Anteroposterior
ASA: American society of anesthesiologists
BMI: Body mass index
CI: Confidence interval
COPD: Chronic obstructive pulmonary disease
CVA: Cerebrovascular accident
DAA: Direct anterior approach
EQ-5D: EuroQol 5 dimensions questionnaire
GEE: Generalized estimating equations
GRSC: Global rating of change scale
HOOS: Hip disabilities and osteoarthritis outcome score
ICER: Incremental costeffectiveness ratio
ICIQ-SF: International consultation of incontinence questionnaire-short form
IPAQ: International physical activity questionnaire
KL: Kellgren and Lawrence
MIS THA: Minimally invasive total hip arthroplasty
MRI: Magnetic resonance imaging
NYHA: New York heart association
PASS: Patient acceptable symptom state
PROM: Patient-reported outcome measure
QALY: Quality adjusted life years
ROC: Receiver operating characteristic
SCT: Stair-climb test
SPSS: Statistical package for the social sciences
SPWT: 4x10m Self-paced walk test
THA: Total hip arthroplasty
TUG: Timed up and go
WHODAS II: World Health Organisation Disability Assessment Schedule II
