# Supplementary material for: The design of a randomised controlled trial to evaluate the (cost-) effectiveness of the posterolateral versus the direct anterior approach for THA (POLADA – trial)
Source: BMC Musculoskelet Disord. 2016 Nov 15;17:476. doi: 10.1186/s12891-016-1322-2 (PMC5111237; doi:10.1186/s12891-016-1322-2)
Supplement: Additional file 1: — Fast-track protocol. (DOCX 14 kb) [file 12891_2016_1322_MOESM1_ESM.docx]

Additional file 1. Fast-track protocol

| Preoperative evaluation   - Education about the protocol - Assessment of pain, mobilization, nutrition and living situation - Control of hemoglobin |
| --- |
| Postoperative management   - No drains are used - No urinary catheters, unless   - BMI≥30   - Urinary infection or urine incontinence   - Urinary retention >400 mL - Control of micturition and defecation - Mobilization 2-6 hours postoperatively, provided that the patient is not nauseous and the pain is under control - Mobilizing with physical therapist two times a day until discharge |
| Medication   - 1 hour preoperatively   - Paracetamol 1000mg   - Gabapentin 600mg   - Naproxen 500mg - 30-45 min preoperatively   - Dexamethasone 0.15mg/kg - 15-60 minutes preoperatively   - Cefazolin 2000mg - Perioperatively   - Bupivacain 0.5% 10mg   - Tranexamic acid 1000mg iv (<100kg), 1500mg iv (>100kg)   - Ondansteron 4mg iv   - Propofol 0.1-2 ug/kg/min   - Ketanest 15 mg iv - Postoperatively   - Paracetamol 1000mg 4d1   - Gabapentin 300mg 1d1 (06.00) and 600mg 1d1 (18.00)   - Naproxen 500 mg 2d1   - Oxynorm 10mg 1d1   - Oxycodon 5mg when pain score ≥ VNRS 4 |
| Discharge criteria   - Medical   - Pain is under control   - Vital parameters are normal   - Micturition/defecation/nutrition are normal   - Medication is prescribed   - Aftercare is taken care of (if necessary) - Physical therapy   - Ability to ambulate with walking aid(s)   - Ability to walk up and down a staircase   - Rules and exercises are fully understood |

*Abbreviations:* BMI = Body Mass Index; VNRS: Verbal Numeric Rating Scale
